# Selecting Presuppositions in Conditional Clauses. Results from a Psycholinguistic Experiment

**DOI:** 10.3389/fpsyg.2015.02026

**Published:** 2016-01-12

**Authors:** Filippo Domaneschi, Elena Carrea, Carlo Penco, Alberto Greco

**Affiliations:** University of GenoaGenoa, Italy

**Keywords:** presuppositions, conditional clauses, update semantics, context change potential, cognitive load

## Abstract

In this paper, we propose an experiment concerning presupposition selection in conditional sentences containing a presupposition trigger in the consequent. Many theories claim that sentences like *if p*, *q**q*'—where *q* is the presupposition of the assertive component *q*'—have unconditional presuppositions, namely, they simply project *q*. Other theories suggest that these kinds of conditional sentences project conditional presuppositions of the form *if p*, *q*. Data collected suggest two results: (i) in accordance with other experiments (by Romoli), dependence between the presupposition *q* and the antecedent *p* favors the selection of a conditional presupposition *if p*, *q*. (ii) presupposition selection in conditional sentences with a trigger in the consequent is affected by speakers' cognitive load: if speakers are highly cognitive loaded, then they are less disposed to select a conditional presupposition. We conclude by arguing that cognitive load represents a key factor for the analysis of linguistic and philosophical theories of context.

## Presuppositions, context set, and compositionality

According to the standard semantic framework, the common ground is the set of propositions that participants in a conversation mutually assume to be taken for granted (Stalnaker, 2002). In this view, the common ground determines the *context set*, that is, the set of possible worlds in which all the propositions that form the common ground are true (Heim, 1983, 1992).

According to this standpoint, the meaning of a sentence is modeled via its *context change potential* (CCP): an instruction to update the context with new information with the effect of producing a new *updated* context as result. For instance, if the context *c* corresponds to the set of possible worlds in which “I have a sister,” “Konstanz is in Europe,” and “Today is Monday” and, in this context, a speaker utters the sentence “I've bought a new car” (ϕ), then the assertion of this sentence in the context *c* (i.e., *c*+ϕ) produces, as a result, a context *c'* that corresponds to the set of possible worlds in which “I have a sister,” “Konstanz is in Europe,” “Today is Monday,” and “I've bought a new car.”

From this perspective, presuppositions put requirements on the context: if ψ is a presupposition of ϕ, then *c* + ϕ is defined only if *c* ⊆ ψ. For example, the sentence “My car is red” can only be uttered in contexts that entail the presupposition “I have a (unique) car.” A sentence's CCP, therefore, is the extent to which the sentence changes the context in which it is uttered to produce a new context, assuming that the new context accepts as true not only the sentence itself but also the presupposition of the uttered sentence. In general, CCP may be defined as a partial function from contexts to contexts: a sentence ϕ can only be uttered in a given class of contexts and brings about a new class of contexts as result^1^.

In order to provide an explanation of how the context changes in the course of a conversation, different dynamic semantic theories have proposed formal representations of language structure aimed at modeling the growth of information in the processing and development of a discourse. Overall, this aims to provide a solution to the traditional problem of the compositionality of meaning, that is, an explanation of how the meaning of compound sentences depends systematically on the meaning of their constituents and on the logical operators in use (e.g., negation ¬ϕ, conjunctions ϕ ∧ φ, disjunctions ϕ ∨ φ, and conditionals ϕ → φ).

In this respect, for many years, linguists and philosophers have been interested in the so-called “presupposition projection problem” (Heim, 1983, 1992; Geurts, 1999; Beaver, 2001; Schlenker, 2008, 2009; Singh, 2008; Kripke, 2009), that is, the problem of the compositionality of presuppositions, how complex sentences inherit their parts' presuppositions. This paper deals in particular with one of the most-discussed topics in this field of research: the Proviso Problem, the problem of the projection properties of conditional sentences with a presupposition trigger in the consequent.

## The proviso problem

The Problem concerns the projection properties of a specific case of composed clauses, conditional sentences that contain a presupposition trigger^2^ in the consequent (CpC); schematically, *if p*, *q**q'* (where *q* is a presupposition triggered by the assertive component *q'*). This core problem, the Proviso Problem (Geurts, 1996), has been widely discussed in recent literature (see for instance, Beaver, 2001; Singh, 2008; von Fintel, 2008; Schlenker, 2010; Chemla and Schlenker, 2011). The discussion has generated two different kinds of answers.

On the one hand, several theories—mainly taking Discourse Representation Theory (DRT) as a framework—claim that sentences of the type *if p*, *q**q'* have mainly unconditional presuppositions, namely, they simply project *q* (e.g., Gazdar, 1979; van der Sandt, 1992; Geurts, 1999). It is, in fact, intuitive that, in several cases, the presupposition projected by a CpC is unconditional; for instance, it is the case in the following utterance (quoted in Geurts, 1999).

(1) If John hates sonnets then *his wife* does so, too.
  (1a). John has a wife

(1) projects the unconditional presupposition, (1a). These theories do not exclude the possibility of deriving a conditional entailments, of the form *if p*, *q*, but they claim that the unconditional presupposition is the default reading, since it is the result of the universal preferencing of global over local accommodation. This is because, while the unconditional reading is derived as a presupposition, the conditional reading is inferred as an entailment. In other words, a sentence of the form *if p*, *q**q'* can be represented in at least two ways in terms of discourse structure.

- In the first reading, the presupposition *q* is globally resolved, that is, it is not represented in the utterance structure but in the global discourse structure, and the result is *q* *and if p, then q'* which captures the unconditional presupposition *q*_._
- In the second reading, *q* is locally represented and the result is *If p, then* *q* *and q'*, which entails the conditional sentence *if p, then* *q*^3^.

In the latter view, the local resolution of the presupposition is supposed to be possible only in contexts where it is supported by a “bridging inference” of the form *if p then it's usual that* *q* based on world knowledge (Geurts, 1999; Piwek and Krahmer, 2000). For example, the local resolution that leads to the conditional entailment (2a) in the case of the sentence (2) is allowed by the bridging inference, “If Mark is a Professor, then it's usual that he has students.”

(2) If Mark is a Professor, then his students love him.
  (2a). If Mark is a Professor, then he has students.

On the other hand, competing theories, traditionally known as “satisfaction theories,” whose subscribers are often also supporters of dynamics semantics^4^, predict that CpC always project conditional presuppositions of the form *if p*, *q* and derive the unconditional presupposition in different ways depending on the versions of the theory (e.g., Heim, 1983; Beaver, 2001; Singh, 2007; van Rooij, 2007; Chemla, 2009). A seminal idea proposed by Heim (1983) has been developed within the framework of update semantics: when a context *c* does not satisfy or does not admit an assertion of *if p, then* *q**q'*, the repair of the context is driven by the instruction c[*if p, then* *q*][*if p, then* *q**q'*]. For example, informally, to update the context *c* with the information conveyed by (2), it is first necessary to update the *context set* with the information (2a).

Let us now consider the following examples (quoted in Pérez-Carballo, 2009).

(3) If Paul is not tired, then he will read his Bible tonight.
  (3a). If Paul is not tired, then he has a Bible.
  (3b). Paul has a Bible.
(4) If Paul is a devout Catholic, then he will read his Bible tonight.
  (4a). If Paul is a devout Catholic, then he has a Bible.
  (4b). Paul has a Bible.

As pointed out by Pérez-Carballo (2009), intuitively, (3) seems to project the unconditional presupposition (3b), while (4) seems to project the conditional presupposition (4a). A possible explanation for that diversity is that, since “the only difference between the two examples is the antecedent clause, the antecedent clause must play an important role in the present phenomenon” (Romoli et al., 2011; p. 593). In particular, the dependence of the antecedent on the presupposition of the consequent seems to play a crucial role in the Proviso Problem^5^. This dependence seems specifically to affect the selection of conditional and unconditional presuppositions, which is traditionally identified by Singh (2007, 2008) and Schlenker (2011, p. 2) as the “Selection Problem.” This problem needs to be distinguished from the “Strengthening Problem,” that is, the question of which mechanisms generate these presuppositions.

In what follows, we focus on the Selection Problem, with a view to grasping whether and when conditional and unconditional presuppositions are selected depending on the relation between the antecedent and the consequent of CpC, specifically, depending on the bridging relation between the presupposition of the consequent and the antecedent of the conditional. In the last decade, the presupposition projection problem has been the subject of several experimental studies but, to our knowledge, no work has been directly aimed at evaluating the relationship between presupposition projection and working memory. Our central goal, besides the confirmation or disconfirmation of previous experimental results, is to study the cognitive load factor in relation to the presupposition selection in CpC. The importance of this aspect in the experimental investigations of ordinary language is due to the widely accepted idea that *the greater the extent to which people are cognitively loaded, the greater their difficulty in processing certain information*. Work on the relationship between cognitive load and conditional reasoning or processing conditional sentences has already produced interesting results, such as Toms et al. (1993), Markovits et al. (2002), Meiser et al. (2001), Capon et al. (2003). Our experiment uses this basic idea, generating different levels of cognitive load to assess whether this affects the subject's understanding or grasping of a conditional or unconditional presupposition in CpC. We might say, therefore, that the general question at stake here concerns the compositionality of presuppositions: what factors affect the selection of either a composed or a simple presupposition?

## An experimental study

The aim of this experimental study is to test three hypotheses about presupposition selection in CpC. The first two hypotheses have been already investigated by Romoli et al. (2011), although, here, we propose a different experimental design, which is also required to test the third hypotheses.

- The conditional presupposition *if p*, *q* is selected more frequently than the unconditional presupposition *q*.
- The conditional presupposition *If p*, *q* is more likely to arise when the presupposition *q* in the consequent is dependent on the antecedent *p*.
- Speakers' cognitive load affects the selection of the presupposition (conditional or unconditional).

Our experiment has been designed to measure the frequency of selection of conditional and unconditional presuppositions. The preponderance of either conditional or unconditional presuppositions, however, does not directly constitute something that can decide between the two approaches: DRT vs. satisfaction theories. In fact, the two approaches each predict that both conditional and unconditional presuppositions can arise and neither concerns itself directly with predicting the frequency of each kind of presuppositions. For one approach, the *default* reading is the conditional presupposition, for the other, the unconditional. The main purpose of this paper, therefore, is to take a first step toward a better understanding of the main factors that affect the frequency of conditional vs. unconditional presuppositions.

In the experiment, participants were required to perform two tasks simultaneously. The main task consisted of listening to a short recording, containing sentences of the type *if p*, *q**q'* and, after that, choosing one sentence that best fits with the recording, from a list of four alternatives. The second task, included in Trials 1 and 3 of the experiment, was to remember two geometrical Figures during the first part of the main task (listening to the recordings). Trials 1 and 3 included the Interference condition, while Trials 2 and 4 included the Simple condition, without interference in the main task.

### Pre-experiment

Two kinds of target items (sentences of the form *p*, *q**q'*) were needed for the experiment: Dependent items, in which the presupposition *q* in the consequent was strongly related to the antecedent content *p*, and Independent items, in which there was no dependence between *p* and *q*. In order to select appropriate items, a questionnaire was created (completed by pencil and paper) similar to the one used by Romoli et al. (2011).

The participants in the pre-experiment were 23 students (15 women, 8 men) from the University of Genoa. They were recruited for course credit. Their ages ranged between 21 and 32 (*M* = 23.95; *SD* = 3.27). All participants were native Italian speakers. Informed consent was obtained.

In the questionnaire, sentences, each followed by a question, were presented to participants; for instance, “Lucy has a dog. Does that make it more likely that she has a leash?” The task was to give an assessment on a 5-point Likert scale, from 1 (much less likely) to 5 (much more likely). The questionnaire included 39 items. The five items—four tests, plus one instruction trial—with the highest score were chosen as target Dependent items, while the five items with the scores closest to the neutral 2.5 point were chosen as target Independent items.

### Participants in the main experiment

Participants in the main experiment were 30 students (14 women, 16 men) from the University of Genoa. None had previously taken part in the pre-experiment. They were recruited for course credit. Their ages ranged between 20 and 31 (*M* = 25.8; *SD* = 2.94). All participants were native speakers of Italian. Informed consent was obtained.

### Stimuli

We created 5 recordings concerning fictional crimes^6^. Every sentence of each recording was read by different female and male voices. We used a whodunit subject in order to encourage participants to be more attentive to details, as if they were detectives^7^. The sentences that constituted the stories were in fact seemingly unrelated and participants had to interpret them as clues to be collected and interpreted, as if they were detectives. Each recording comprised between 51 and 66 words (an average of 58). Three conditional sentences, with balanced order, were included in each recording: (i) a Dependent target conditional sentence, (ii) an Independent target conditional sentence, (iii) a distractor conditional sentence. Dependent and Independent target sentences were selected on the basis of the results obtained in the pre-experiment. All the target conditional sentences activated a presupposition in the consequent via the presence of a definite description. For instance, the Recording 1 ran as follows.

The thief came into the house during the night. Luke's father is the owner of the house. *If Luke is a writer, then his book is sold at the bookshop* [Dependent target]. Mud stains were found on the carpet in the living room. *If the thief came into the house passing through the garden, then he should have left footprints* [distractor]. *If Luke is tall, then he will tell one of his jokes to the cops* [Independent target].

Two sets of four sentences were connected to each recording, a Dependent set and an Independent one. In each set, there were included:

- a sentence [C] corresponding to the conditional presupposition of the target sentence (Dependent or Independent);
- a sentence [U] corresponding to the unconditional presupposition of the target sentence (Dependent or Independent);
- a conditional filler sentence reporting wrong or unmentioned information about general content of the recording [Fc];
- a unconditional filler sentence reporting wrong or unmentioned information about general content of the recording [Fu].

For example, the Dependent set of sentences related to Recording 1, printed above, was:

- [C] If Luke is a writer, then he has written a book.
- [U] Luke has written a book.
- [Fc] If the house is beautiful, then the thief came into the house singing.
- [Fu] The thief came into the house singing.

The Independent set included the following four sentences:

- [C] If Luke is tall, then he knows some jokes.
- [U] Luke knows some jokes.
- [Fc] If the thief wore slippers, then he had mud on his pants.
- [Fu] The thief wore slippers.

**Figure 1:**
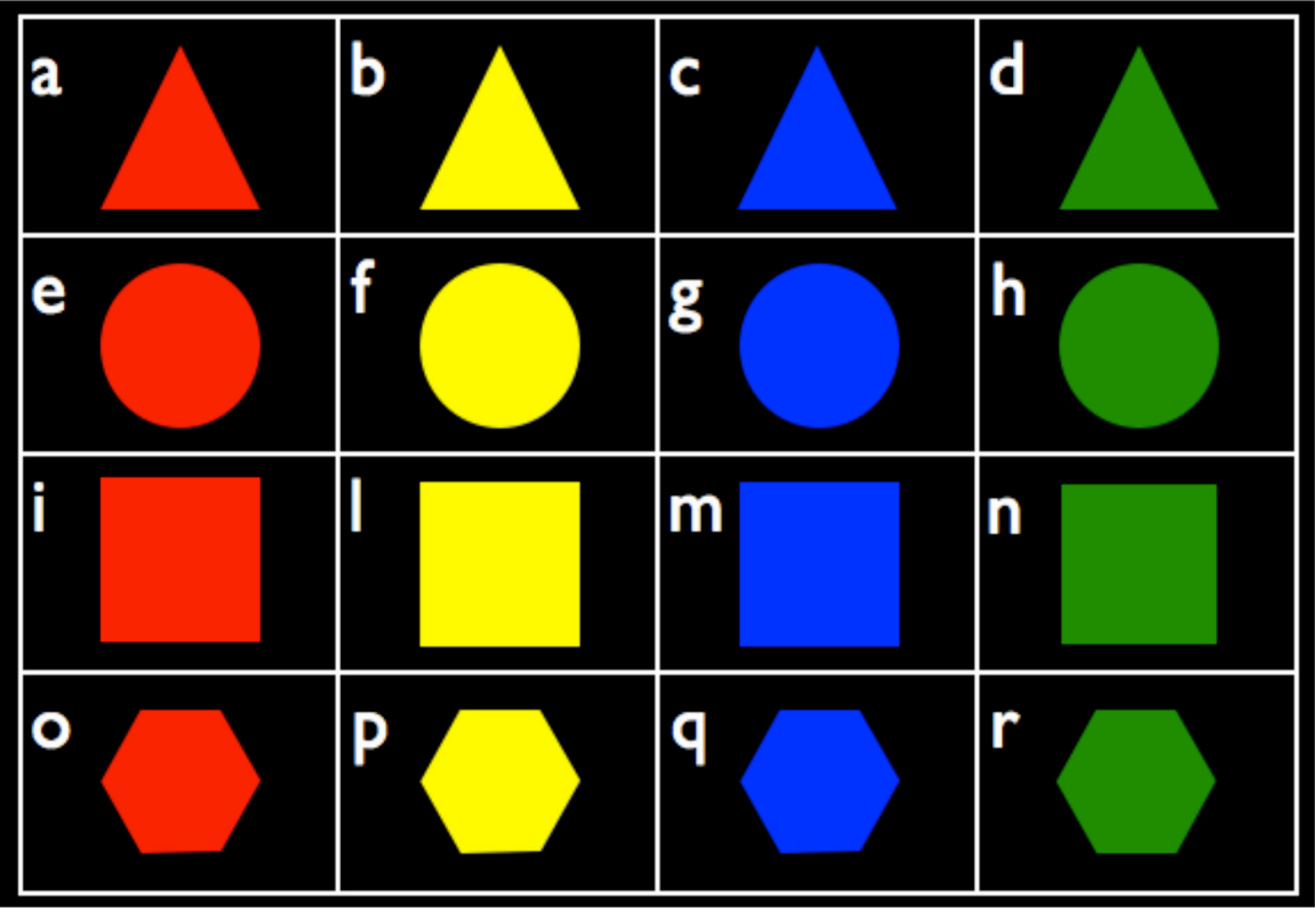
**The sixteen polygons used as stimuli to load participants' working memory**.

Sixteen polygons were created (Figure 1) by combining four shapes—triangle, square, hexagon, circle—with four colors—red, green, yellow, blue. These figures were used to load participants' working memory during the execution of the first part of the main task, namely, listening to recordings.

### Procedures

The study was conducted in a laboratory setting. Instructions, stimuli, response recording, and data collection were controlled by a laptop computer running E-Prime® 1.1. Participants sat approximately 50 cm from the display, in a separate room. The lighting in the room was normal. Only a keyboard (no mouse) was available for responses.

The experiment included four trials for each participant. Only Trials 1 and 3 included the second task about geometrical figures. Trials 1 and 3 represented therefore the Interference condition, while Trials 2 and 4 represented the Simple condition, without interference.

**Figure 2:**
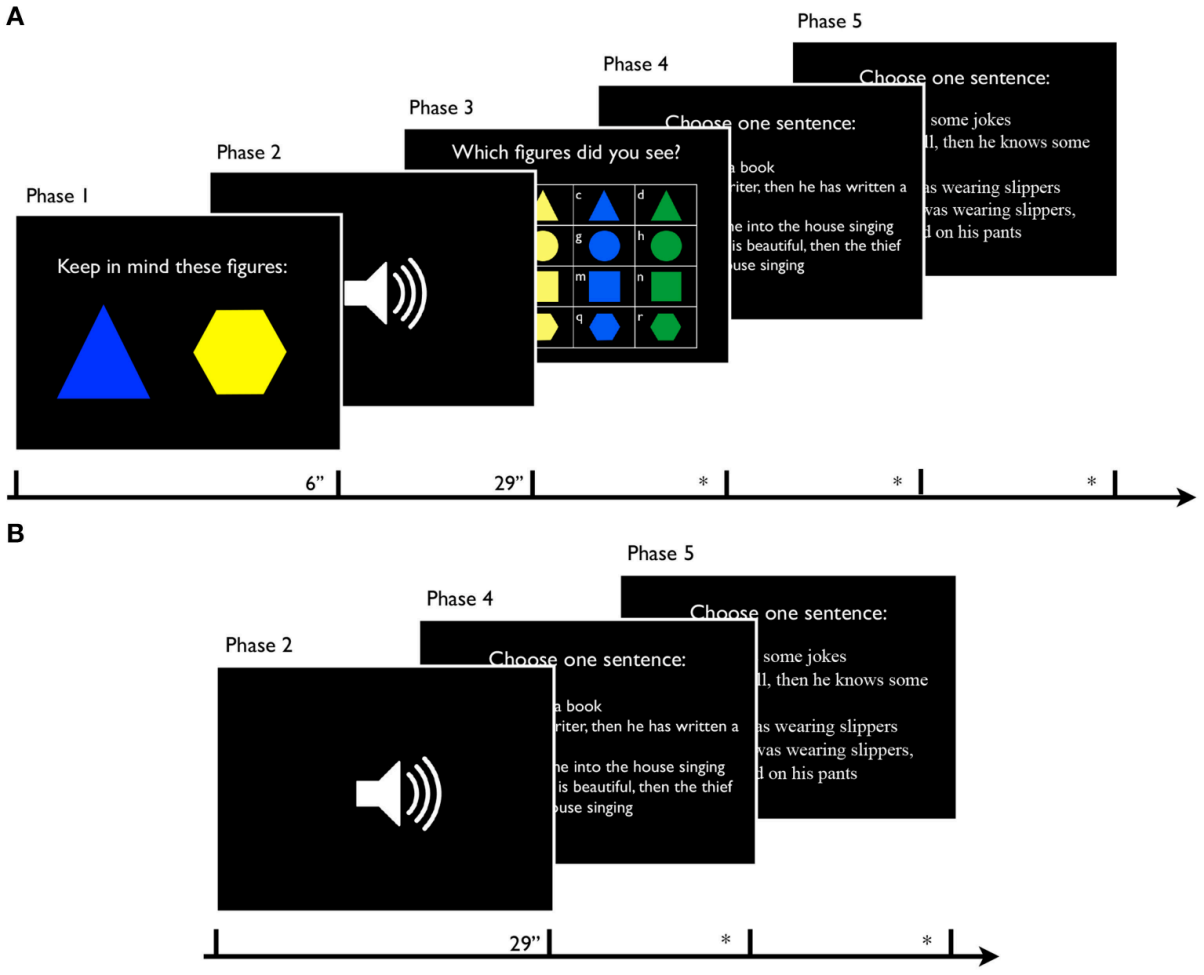
**Screenshots of each phase in Interference condition (A) and Simple condition (B) trials**.

The Interference condition trials consisted of the following phases (Figure 2A).

1. Two geometrical figures were shown on the screen for 6 s.
2. Participants listened to an audio recording. This phase lasted 29 s.
3. Participants were required to indicate which polygons they had observed during Phase 1. This step was repeated twice: on the first screen, they indicated the first polygon seen at the beginning of the task (e.g., blue triangle); the same screen was then presented for a second time in order to let them indicate the second polygon (e.g., yellow hexagon).
4. The Dependent set was shown to participants. The task was to choose one of the four sentences regarding the recordings, following their intuition. No time limit was introduced but participants were required to select the sentence as quickly as possible.
5. Phase 4 was repeated by showing the Independent set.

In the Simple condition, the trials (Figure 2B) included only Phases 2, 4, and 5.

By way of instruction, the task was explained to participants by using a sample trial. The trials' order did not change during the experiment, while the presentation order of the Dependent and Independent sets and the presentation order of the four sentences within each set were randomized for every participant.

The figures used to load participants' working memory were chosen randomly but kept fixed for each trial (e.g., Recording 1 was always presented with a green triangle and a red hexagon). This was in order to show participants equally difficult combinations of figures.

### Expectations

Our expectations were as follows.

1. For Romoli et al. (2011), participants were led to select more conditional presuppositions *if p*, *q*, then unconditional ones, both in case of Dependent target sentences and of Independent target sentences. In particular, this pattern was expected for the Simple condition, since it was similar to Romoli et al.'s design, which did not include any interference task.
2. For Romoli et al., the conditional presupposition *If p*, *q* is more likely to arise when the presupposition *q* in the consequent is dependent on the antecedent *p*. Since we used a within-subject design instead of the between-subject design adopted by Romoli et al. we aimed to analyze the effect of the dependence for the very same participant on the selection of the presupposition, in order to provide a further confirmation of the dependence hypothesis.
3. We expected the cognitive load factor might affect participants' behavior in the selection of the conditional presuppositions *if p*, *q* in both the Simple and the Interference conditions. More precisely, since the processing of conditional sentences seems to depend more on the resources available in the working memory than the processing of unconditional (Toms et al., 1993), it is reasonable to assume that processing and representing a conditional presupposition is likely to be more cognitively demanding than doing the same for an unconditional presupposition. Hence our expectation was that participants would more frequently select an unconditional presupposition [U] instead of a conditional one [C] in an interference condition, where they have limited resources available for processing the conditional presupposition.

### Results

The data from two participants were excluded from the analysis because of an interruption in task performance. Considering the second task, with regards to memorizing geometrical figures, the mean of correct answer was 0.88 (*SD* = 0.32). Every participant reached at least 50% of correct answer and thus none of them were excluded from the analysis.

**Figure 3:**
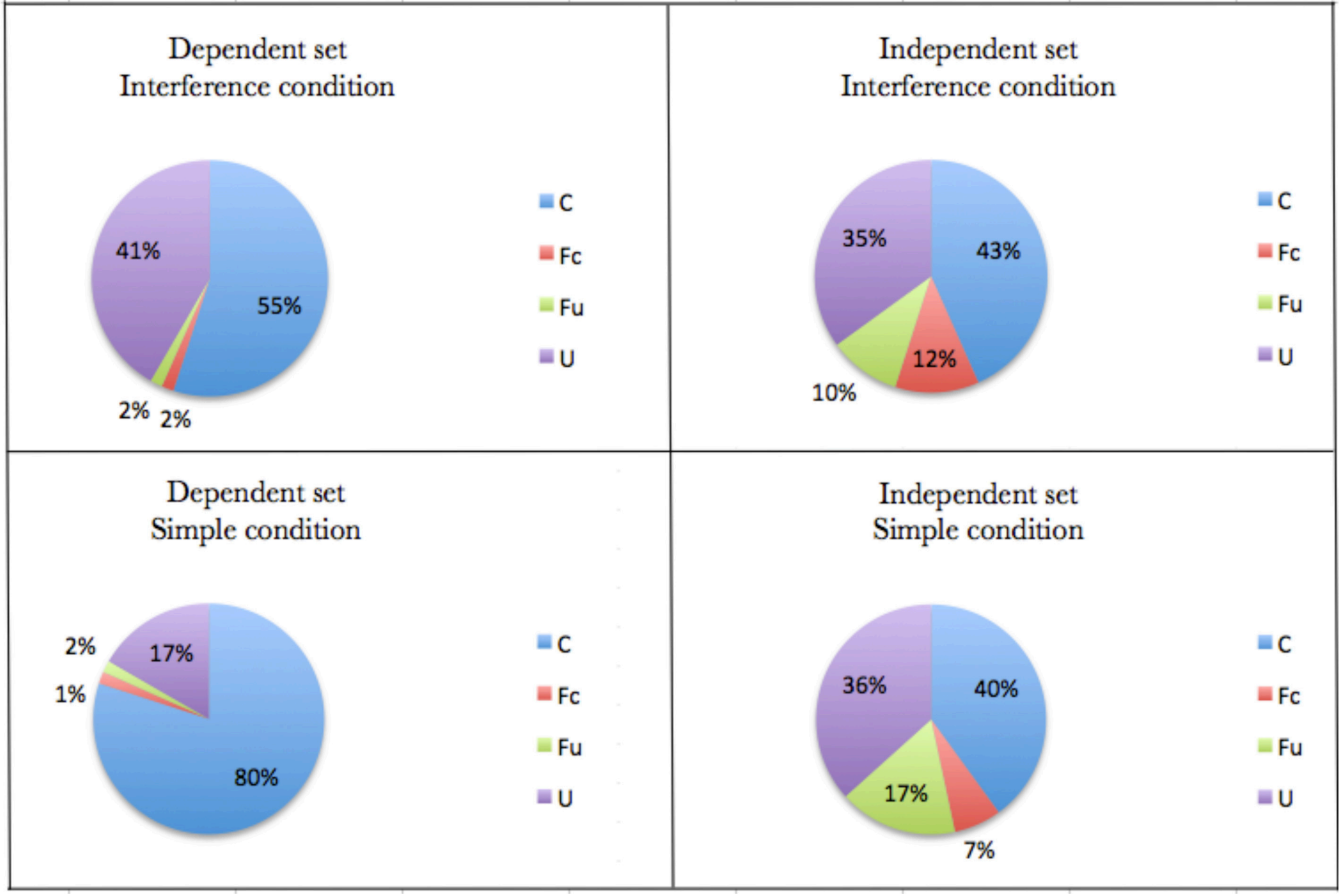
**The general results graphically summarized in percentages**.

The general results are reported in Table 1 and graphically summarized in Figure 3. Considering Expectation 1 above, we analyzed the percentage of conditional presupposition selection [C] with respect to the percentage of unconditional presupposition selection [U] in both conditions. The results were:

- **Result 1A**: The percentage of conditional presuppositions [C] was significantly higher than unconditional presupposition [U], using results for Dependent set plus Independent set in the Simple condition (Wilcoxon Signed Rank: *W* = 29.5, *p* < 0.001).
- **Result 1B**: The comparison between [C] and [U], using results for Dependent set plus Independent set, did not result in a statistical difference under the Interference condition.

**Table 1:** **The general results of the experiment under the two conditions (Interference, Simple) and the two sets of answers (Dependent, Independent) reported as total frequency of choice**.

	**Dependent set**	**Independent set**	**Total**
Interference cond.	60	60	120
C	33	26	59
Fc	1	7	8
Fu	1	6	7
U	25	21	46
Simple cond.	60	60	120
C	48	24	72
Fc	1	4	5
Fu	1	10	11
U	10	22	32
Total	120	120	240

*Results concern conditional presuppositions [C], conditional fillers; [Fc], unconditional fillers; [Fu], unconditional presuppositions [U]*.

The data seems to be in line with Expectation 1 and those produced under the Simple condition were consistent with Romoli et al.'s results.

Considering expectation (2), data collected seemed to show that:

- **Result 2A**: the percentage of conditional presuppositions [C] selected in the Dependent set was significantly higher than the percentage of [C] in the Independent set (Wilcoxon Signed Rank: *W* = 6.5, *p* < 0.001).
- **Result 2B**: this pattern, in result 2A, did not emerge under the Interference condition.

Finally, considering Expectation 3, data collected seem to show that:

- **Result 3A**: the percentage of [C] selected in the Interference condition was lower than the same percentage in the Simple condition with regards to the Dependent set (Wilcoxon Signed Rank: *W* = 67, *p* < 0.05).
- **Result 3B**: this effect was not observed in the Independent set.

## General discussion

The general goal of this experiment was to investigate the Selection Problem in presuppositions projection in conditional sentences with a presupposition trigger in the consequent. In particular, our experiment was aimed at evaluating the dependence hypothesis considered by Romoli et al. (2011) and the role played by participants' cognitive load.

Data collected showed two results.

(1) Participants, in general, selected the conditional presuppositions more frequently than the unconditional presuppositions in processing CpC, as reported in **Result 1A**.

This first result is sympathetic to Conclusion (i), proposed by Romoli et al., according to which conditional presuppositions are more likely to be selected than unconditional presuppositions. This conclusion was confirmed by the data we have collected under the Simple experimental condition^8^. Therefore, **Result 1A** seems to support the central thesis of satisfaction theories that all CpCs project mainly conditional presuppositions of the form *if p*, *q*.

(2) Participants selected the conditional presuppositions more frequently when there was dependence between the antecedent of the CpC and the presupposition activated by the trigger in the consequent, as reported in **Result 2A**.

Reconsidering the Simple condition, **Result 2A** seems to be compatible both with satisfaction theories and with theories which predict that CpC mainly project unconditional presuppositions. In fact, the former theories claim that, in cases of CpC, conditional presuppositions are selected most of the time, hence the conditional presupposition *If p*, *q* is more likely to arise when the presupposition *q* in the consequent is dependent on the antecedent *p*. According to the latter theories, even if the unconditional presupposition is the preferred reading in cases of CpC, when there is dependence between the antecedent of a CpC and the presupposition triggered in the consequent, speakers are supposed to select a conditional presupposition.

**Result 2A**, therefore, coheres with the idea, proposed by Romoli et al. (2011), that the conditional presupposition *If p*, *q* is more likely to arise when the presupposition *q* in the consequent is dependent on the antecedent *p*. Moreover, since we used a within-subject design instead of the between-subject design adopted by Romoli et al., we did not analyze data collected from different participants assigned to two different conditions. Rather, we analyzed the effect of dependence, for the same participant, on the selection of the presupposition, where dependence was the only manipulated variable. Hence, this analysis allows us to support a stronger claim: the dependence between the antecedent and the presupposition in the consequent of a CpC has a relevant effect in the selection of the presupposition.

To sum up, **Results 1A** and **2A** support the idea that sentences of the form *If p*, *q**q'* mainly project conditional presupposition as *If p*, *q* and even more so if there is dependence between *p* and *q*^9^.

The second purpose of our experiment was to explore the effect of participants' cognitive load. To this end, data collected seem to suggest that:

(3) the same participant, if highly cognitively loaded, selected conditional presuppositions less frequently then in the case of low cognitive load, as occurred under our Simple condition (see **Result 3A**).

Our statistical analysis seems to show that, in the Dependent set, where participants were supposed to project conditional presuppositions (as shown by **Result 2A**), the very same participant, if highly cognitively loaded, might project an unconditional presupposition instead of the conditional presupposition that she probably would have projected if she had had more cognitive resources available. Considering the percentages of conditional and unconditional presuppositions selected within the set of dependent targets, data collected suggest that, under the Interference condition, the percentage of conditional presuppositions projected decreases, while the percentage of unconditional presuppositions increases.

**Result 3A** allows us to claim that, to a certain extent, together with the dependence between the antecedent and the presupposition in the consequent, speakers' cognitive load is a relevant factor that affects the selection of the presupposition in CpC. One explanation for this result might be that highly cognitive loaded speakers are less disposed to select a conditional presupposition since processing the mental representation corresponding to a composed sentence, and, in particular, to a conditional sentence, requires more cognitive effort than is the case for a simple (i.e., unconditional) sentence. Toms et al. (1993), for example, have argued that mistakes in conditional reasoning are related to working memory. In particular, conditional reasoning seems to require a surplus in working memory that, in turn, requires support from the central executive. In conditional representations, the higher the number of models required, the higher the cognitive effort involved (Barrouillet and Lecas, 1999; Johnson-Laird, 2001). Hence, the limited available resources under the Interference condition might have affected the selection by changing a conditional answer [C] in an unconditional answer [U]^10^.

Some final considerations concern the set of independent targets in our experiment. First of all, we have shown in **Result 2A** that independent conditional presuppositions have been selected significantly less frequently than dependent conditional presuppositions under the Simple condition. Thus, the percentage of independent conditional presuppositions under the Simple condition was close to the percentage of unconditional presuppositions. This result might be explained by assuming that. in the Independent set, since there was no sort of bridging inference connecting the content of the antecedent and the content of the consequent of the conditional presupposition of the CpC, participants have treated independent conditional presuppositions in the same way, as if they were independent unconditional presuppositions. In this case, in other words, the conditional presuppositions have been evaluated as equally available by the participants so that, in terms of percentage, they have been equally selected in the course of the experiment.

Secondly, a comparison of **Results 2A** and **2B** shows that the percentage of conditional presuppositions selected in the Dependent set decreases from the Simple condition to the Interference condition, while the percentage of conditional presuppositions selected in the independent set does not change significantly from the Simple condition to the Interference condition. These data seem to support the idea that, while the cognitive load factor affects the selection of the presuppositions in the Dependent set, it does not seem to have an effect on the selection in the Independent set. The data, therefore, suggest that the cognitive load factor affects the selection of the presupposition of a CpC only when there is a dependence between the antecedent of the conditional and the content of presupposition triggered in the consequent: if the dependence holds, and the speaker is highly cognitively loaded, then she seems to be less disposed to select a conditional presupposition. This effect of the cognitive load factor on the presupposition selection in a dependent CpC might be explained by the bridging inference that supports the dependence. The reason may be that, under the Interference condition, participants had few cognitive resources for performing the main task, given that part of their cognitive resources were used in the second task (i.e., memorizing geometrical figures). Since processing the dependent conditional implied computing the bridging inference (e.g., computing that “If Paul is a devout Catholic, then he will read his Bible tonight” implies computing the bridging inference “If someone is a devout Catholic, then he or she usually has a Bible”), the interference of the second task under the Interference condition affected the selection of the dependent conditional presuppositions. The reason seems to be that the remaining resources for performing the main task were not sufficient for computing both the content of the conditional presuppositions and the bridging inferences, with participants consequently selecting less dependent conditional presuppositions under the Interference condition than in the Simple condition. Conversely, processing independent conditionals does not require computing any bridging inference; hence, under the Interference condition, the remaining resources for performing the main task were sufficient for selecting the conditional presuppositions, meaning that, under the Interference condition, participants selected independent conditional presuppositions as often as under the Simple.

To conclude, data collected support the idea that two relevant factors affecting presupposition selection in the Proviso Problem are (i) dependence between the antecedent of a CpC and the presupposition triggered in the consequent and (ii) speakers' cognitive load.

While presuppositions have, for a long time, been rather unexplored as a topic in the field of experimental pragmatics, in the last years, a new wave of studies (Schwarz, 2014; Domaneschi, 2015) have suggested that, while presuppositions are typically considered background meanings, expected to be processed automatically, the actual processing seems to involve a large chunk of the cognitive resources available to the language users, which affects the understanding of different kinds of presuppositions.

It was expected that different factors would affect the cognitive demand of processing a presupposition. This paper has attempted to show that compositionality (i.e., conditional vs. unconditional presuppositions) is one of these crucial factors.

## Some final remarks concerning the cognitive load factor

Cognitive context, the set of presuppositions assumed to be taken for granted by the participants in a conversation, has been widely discussed since the debate on informative presupposition (Gauker, 1998, 2008; Stalnaker, 1998, 2002; von Fintel, 2008) and the distinction between passive and active (or local) context (Kripke, 2009; Schlenker, 2009, 2011). On this background, the notion of cognitive context (and of contextual felicity) is still a working theoretical notion at the boundary between semantics and pragmatics and is useful for treating the pragmatic phenomenon of accommodation (von Fintel, 2008; Tonhauser et al., 2013). However, while the relevance of different cognitive loads on processing conditionals is a usual topic in psychological discussion, linguistic and philosophical theories of presuppositions have usually bypassed the problem. However, doing so runs the risk of treating the concept of cognitive context (perhaps including the distinction between passive and active context discussed by Kripke, 2009) without considering that *which* context is shared—which presuppositions are activated—may depend on the kind of cognitive effort required in a conversation. Without taking into account the impact of the cognitive effort behind selecting certain linguistic content in a context (e.g., presuppositions), we might overlook or misunderstand some experiments' results and, consequently, be unable to select the right competing theory.

One of the problems with theories of communication based on classical linguistic and philosophical theories is that they sometimes depend on hypotheses concerning how hearers should react or what they should understand to have been discussed *without* taking into account different possible scenarios arising from hearers' different cognitive loads. In a previous work (Domaneschi et al., 2014), we have discussed the role of cognitive effort in detecting presuppositions, showing that some presuppositions (mainly Iteratives and Change of State Verbs, which deal with temporal features) are more difficult to process when a hearer is highly cognitively loaded. The present study, plus Domaneschi et al. (2014) give some provisional methodological suggestions concerning the impact of the role of cognitive load in assessing the plausibility of linguistic and philosophical models: (i) the analysis of the cognitive load factor might reveal that, even if a certain semantic reading of a sentence appears to be like the default, that is, the one determined by the meaning of the clause, language users can opt for a less probable reading that is nevertheless more compatible with their available cognitive resources. Missing this point may affect how the different hypotheses under examination are assessed. (ii) The cognitive context (the set of presuppositions) might change depending not only on the logic of the discourse structure but also on the speakers' cognitive state, namely, the level of cognitive load of participants in the conversation, which affects what kind of presuppositions are selected and, consequently, how the context changes in the course of a conversational exchange. These considerations we propose as hints for future research that might reveal further unexpected results.

## Funding

The research was partly funded by a Ministero dell'Istruzione, dell'Università e della Ricerca (MIUR) research project 20107738C5_008 coordinated by Michele Marsonet and by the Research Project PRA 2012 Presupposition and Cognitive processes coordinated by CP.

### Conflict of interest statement

The authors declare that the research was conducted in the absence of any commercial or financial relationships that could be construed as a potential conflict of interest.

## Footnotes

^1^To be precise, a sentence is only ever *uttered* in a *particular* context, but the same sentence can be correctly used in all contexts in which its presuppositions are true (we are grateful to a referee of Frontiers for making this distinction).

^2^Presupposition triggers are lexical items and syntactic constructions that, if used in an utterance, activate a presupposition. In contemporary debate, there are two major approaches to the problem of triggering presuppositions. In semantic approaches, it is claimed that presuppositions are a particular type of meaning determined by the lexicon (Chierchia and McConnell-Ginet, 2000; Simons, 2001). Other scholars (Karttunen, 1974; Simons, 2001; Abusch, 2010; Schlenker, 2010) have supported a pragmatic view, according to which presuppositions are the result of speakers' inferences, as well as conversational implicatures (Abusch, 2002, 2010). On more specific relations between presuppositions and scalar implicatures see Pistoia Reda (2014).

^3^*See van der Sandt (**1992**) for the seminal idea of the resolution of presuppositions in DRT*.

^4^Recently, Schlenker (2008; 2009) has proposed a more static approach that makes the same predictions as dynamic semantics with regards to the Proviso Problem. Schlenker (2008), for instance, proposes an account of presupposition projection within a classic semantic framework enriched with two pragmatic principles grounded on the Gricean maxim of manner: “Be articulated” and “Be brief.”

^5^The idea that this kind of probabilistic reasoning is relevant to the Proviso Problem is discussed in Beaver (2001); Lassiter (2012), Schlenker (2011), and von Fintel (2008).

^6^The experiment was run in Italian. The original items have been included in the Appendix in Supplementary Material.

^7^The texts of the recordings and some statements presented incongruous information mainly because the independent conditionals (see below) expressed a link between disconnected, independent contents. In order to make these incongruities plausible to the participants, we required them to act as if they were detectives, namely, by considering the information presented in the recordings as disconnected and incoherent clues provided by witnesses. The post-experimental interview revealed no particular difficulties, on the part of the participants, with these incongruities.

^8^Since Romoli et al.'s design did not include a second task generating interference, our Simple condition was more suitable than our Interference condition for comparison with Romoli et al.'s conclusions.

^9^As said before, all the target sentences used in our experiment have been divided into dependent and independent conditionals thanks to the results of the norming pre-experiment. As pointed out by Romoli et al. further research might address the question about what notion of dependence has been used by participants while answering the questionnaire.

^10^One relevant point concerns the reasons why the conditional presupposition is more costly than the unconditional and whether **Result 3A** says something about the satisfaction vs. DRT-like theories. However, data collected do not seem to be strong enough for making previsions concerning the possible mechanisms underlying the processing of conditional and unconditional presuppositions that might be conjoined with different theories of presuppositions. Rather, data collected by this experimental design allows the recognition simply of the cognitive *amount* of selecting presuppositions in CpC: selecting conditional presuppositions seems to be more cognitively demanding than selecting unconditional ones. Further experimental studies should be conducted to investigate the link between processing conditional and unconditional presuppositions in CpC and the mechanisms employed in the competing theories.
