# Supplementary material for: Selecting Presuppositions in Conditional Clauses. Results from a Psycholinguistic Experiment
Source: Front Psychol. 2016 Jan 12;6:2026. doi: 10.3389/fpsyg.2015.02026 (PMC4709410; doi:10.3389/fpsyg.2015.02026)
Supplement: Supplementary file 1 [file DataSheet1.pdf]

Appendix
Selecting Presuppositions in Conditional Clauses.
Results from a Psycholinguistic Experiment.
Filippo Domaneschi1, Elena Carrea1, Carlo Penco1* and Alberto Greco1

Original Italian versions of all the recordings and lists of questions pairs:

Recording 1
Il ladro è entrato in casa di notte. La casa è di proprietà del padre di Luca. Se Luca è uno scrittore, allora il suo libro è un poliziesco. Sono state trovate macchie di terra sul tappeto in ingresso. Se il ladro è entrato dal giardino, allora avrà avuto della terra sotto le scarpe. Se Luca è alto, allora racconterà una delle sue barzellette ai poliziotti.

Questions – Recording 1
Quale fra queste informazioni ti sembra corretta?
A- il ladro è entrato di giorno
B- Luca ha scritto un libro
C- Se Luca è uno scrittore, allora ha scritto un libro
D- Se la casa è di Luca, allora il ladro è entrato di giorno

Quale fra queste informazioni ti sembra corretta?
E- Se Luca è alto, allora conosce delle barzellette
F- Luca conosce delle barzellette
G- Se il ladro aveva gli stivali, allora avrà avuto della terra sotto le scarpe
H- Il ladro aveva gli stivali neri

Recording 2
Guglielmo è stato sorpreso a rubare un portafoglio sull'autobus. Se Guglielmo beve birra allora il suo rastrello è blu. Il controllore ha chiesto subito i documenti a Guglielmo. Se Guglielmo è un professore, allora i suoi studenti sono preparati. Se il controllore è mancino, allora è sceso dalla prima porta dell'autobus.

Questions – Recording 2
Quale fra queste informazioni ti sembra corretta?
A- Se Guglielmo beve birra, allora ha un rastrello
B- Guglielmo ha un rastrello
C- Guglielmo era senza documenti
D- Se il controllore è mancino, allora Guglielmo ha pagato il biglietto

Quale fra queste informazioni ti sembra corretta?
E- Guglielmo ha degli studenti
F- Guglielmo non è stato sorpreso mentre rubava il portafoglio
G- Se Guglielmo è un professore, allora ha degli studenti
H- Se il controllore ha fatto scendere Guglielmo, allora è sceso dall'ultima porta

Recording 3
A Portofino è stato commesso un omicidio. Se l'assassino è un uomo, allora ha utilizzato un pugnale. Se la vittima è un dentista, allora la sua Bibbia è rilegata in pelle. L'omicidio è stato commesso a mezzogiorno in punto. La polizia è arrivata sul luogo alle 13. Se l'assassino è zio, allora porterà i suoi nipoti al parco.

Questions – Recording 3

Quale fra queste informazioni ti sembra corretta?
A- Se l'assassino è zio, allora ha dei nipoti
B- L'assassino ha dei nipoti
C- L'omicidio è stato compiuto a mezzanotte
D- Se l'omicidio è stato compiuto a mezzanotte, allora la polizia è arrivata alle 13

Quale fra queste informazioni ti sembra corretta?
E- Se la vittima è un dentista, allora ha una Bibbia
F- La vittima ha una Bibbia
G- L'omicidio è stato commesso ad Albisola
H- Se la vittima è una donna, allora l'omicidio è stato commesso ad Albisola

Questions – Recording 4
Se c'è il sole, allora la polizia usa le moto. La polizia ha inseguito il rapinatore per un'ora prima di arrestarlo. Se il rapinatore ha un cane, allora ha perso il guinzaglio in giardino. La refurtiva è stata trovata sotto un albero. La refurtiva consisteva per lo più in gioielli d'oro. Se il rapinatore è bravo al computer, allora sua moglie è gentile.

Recording 4
Quale fra queste informazioni ti sembra corretta?
A- Se il rapinatore ha un cane, allora ha un guinzaglio
B- Il rapinatore ha un guinzaglio
C- La refurtiva consisteva in gioielli d'argento
D- Se i gioielli erano d'argento, allora sono stati trovati sotto un albero

Quale fra queste informazioni ti sembra corretta?
E- Se il rapinatore è bravo al computer, allora ha una moglie
F- Il rapinatore ha una moglie
G- La polizia non è riuscita ad arrestare il rapinatore
H- Se la polizia ha arrestato il rapinatore, allora era in auto

Questions – Recording 5
Francesco è stato rapito mentre stendeva i panni in giardino. Se i panni sono di cotone, allora non sono state trovate impronte digitali nell'appartamento. Se Francesco è un giardiniere, allora il suo rastrello è blu. Se il rapitore è simpatico, allora la sua auto è in ottime condizioni. Il rapitore ha chiesto 10.000 euro di riscatto.

Recording 5
Quale fra queste informazioni ti sembra corretta?
A- Se Francesco è un giardiniere, allora ha un rastrello
B- Francesco ha un rastrello
C- Il rapitore ha chiesto 1.000 euro di riscatto
D- Se Francesco è un giardiniere, allora il riscatto è di 1.000 euro

Quale fra queste informazioni ti sembra corretta?
E- Se il rapitore è simpatico, allora ha un'auto
F- Il rapitore ha un'auto
G- Sono state trovate impronte digitali
H- Se i panni sono di cotone, allora sono state trovate impronte digitali
